# Evaluation of smoking cessation interventions for veterans in HIV clinics in the United States: a theory-informed concurrent mixed-method study

**DOI:** 10.1080/21642850.2021.1967159

**Published:** 2021-08-30

**Authors:** Seth Himelhoch, Veronica P. S. Njie-Carr, Amanda Peeples, Crystal Awuah, Amanda Federline, Isabella Morton

**Affiliations:** a College of Medicine, University of Kentucky, Lexington, USA; b School of Nursing, University of Maryland, Baltimore, Maryland, USA; c Education and Clinical Center, U.S. Department of Veterans Affairs Maryland Health Care System, Mental Illness Research, Baltimore, Maryland, USA; d U.S. Department of Veterans Affairs Maryland Health Care System, Education and Academic Affairs, Baltimore, Maryland, USA; e UCLA Semel Institute, Los Angeles, California, USA

**Keywords:** Barriers, enablers, HIV infection, i-PARIHS framework, Veterans Affairs

## Abstract

**Objective:**

Although veterans living with HIV infection are burdened with smoking-related morbidities, few studies have explored theory-informed, evidence-based smoking cessation interventions in the Veterans Affairs (VA) Health System.

**Method:**

In this concurrent mixed-method study, we sought to better understand factors influencing the adoption of existing evidence-based smoking cessation interventions (reminders, telephone quit lines, pharmacological) for veterans in VA HIV clinics. We explored the alignment of the revised Promoting Action on Research Implementation in Health Services Framework (i-PARIHS) with study results.

**Results:**

Nineteen clinicians working at eight HIV clinics in the VA System participated in the study. Seven themes were identified with relative quantitative and qualitative data convergence of clinicians’ perceptions of the importance of integrating evidence-based smoking cessation interventions for veterans with HIV infection.

**Conclusion:**

Identified themes underscore the need for clinicians to provide smoking cessation training, supportive care, and motivate veterans living with HIV infection to quit smoking. Integrating smoking cessation programs into HIV treatment plans in the veteran patient population is critical. Dedicated time to fully implement these efforts will maximize smoking cessation intervention efforts and will yield successful utilization and subsequent patient compliance. Importantly, combination strategies will ensure cessation program impact and sustainability.

**Trial registration:**Netherlands National Trial Register identifier: ntr050..

## Introduction

Smoking exacerbates the HIV disease trajectory with grave consequences including HIV treatment non-adherence (Harris, 2010; Manuel, Lum, Hengl, & Sorensen, 2013; Moscou-Jackson, Commodore-Mensah, Farley, & DiGiacomo, 2014). About 14% of the adult population in the United States (U.S.) smoke and are at risk for the morbidities associated with smoking (Cornelius, Wang, Jamal, Loretan, & Neff, 2020). People living with HIV (PLWH) smoke three times the rate of the general population (Bean, Richey, Williams, Wahlquist, & Kilby, 2016; Ledgerwood & Yskes, 2016) and are associated with AIDS and non-AIDS related morbidity and mortality (Brandt et al., 2017; Kariuki et al., 2015; Monnig et al., 2016; Sigel, Makinson, & Thaler, 2017; West, 2017).

Smoking is particularly prevalent in the veteran population due to factors that incentivize tobacco use such as low tobacco costs on military bases, dedicated time for smoke breaks, social smoking, and smoking as an idle activity or stress reliever (Reisen et al., 2011; Wilson et al., 2017). Ignacio et al. (2018) found that the Veterans Affairs (VA) Health Administration reported high rates of tobacco use in individuals with psychiatric and substance use disorders. Veterans are also disproportionately affected due to traumatic experiences (Center for Disease Control and Prevention [CDC], 2018a; Ignacio et al., 2018). A study conducted from 2010–2015 found that three in ten veterans use tobacco products with the highest type of tobacco use being cigarettes (CDC, 2018a).

The risk of current smoking is increased in veterans living with HIV (VLWH) (Wilson et al., 2017). The Veterans Aging Cohort Study of 2892 patients with HIV from multiple VA infectious disease clinics revealed that as many as 64% of VLWH smoked (Wilson et al., 2017). Another study that investigated tobacco use in male veterans with HIV in the Washington, D.C. area found a prevalence of smoking to be as high as two thirds in comparison to one-fifth of the general population (Reisen et al., 2011); and higher morbidity and mortality in veterans who smoked compared to veterans who did not (Crothers et al., 2009). Although there is knowledge of increased morbidity associated with smoking VLWH, studies suggest that smoking cessation (SC) is less emphasized due to elevated risk of mortality from HIV (Shirley, Kaner, & Glesby, 2013). SC efforts for PLWH in community treatment centers is minimal and brief, with healthcare workers facing barriers such as lack of time, patient resistance, lack of provider self-efficacy to provide SC interventions, and lack of pharmacotherapy (Ledgerwood & Yskes, 2016).

Evidence-based SC interventions are not routinely offered to PLWH (Kariuki et al., 2015; Tesoriero, Gieryic, Carrascal, & Lavigne, 2010). There is critical need to evaluate SC interventions offered to PLWH to inform the utilization of best practices (Pacek & Crum, 2015). An available SC resource offered to VLWH who smoke falls under the National Smoking and Tobacco Use Cessation Program. Smoking cessation medications are made available to smokers interested in quitting at the VA (Ignacio et al., 2018). The VA Health System has been the vanguard in implementing accessible and evidence-based approaches to evaluate and offer SC interventions for veterans who smoke. The VA provides reminders to screen for SC within the electronic medical record, offers free access to telephone-based quit lines, and provides access to evidence-based pharmacological treatment approaches for SC.

Recognizing the high prevalence of smoking among VLWH and the challenges of SC intervention uptake throughout the VA, the aim of this study was to evaluate factors influencing the utilization of evidence-based SC treatment interventions at eight HIV treatment sites within the VA System using a mixed-method approach. The Revised Promoting Action on Research Implementation in Health Services Framework (i-PARIHS) (Harvey & Kitson, 2016) was used to deductively frame the evaluation of SC interventions among VLWH. The constructs include *Innovation, Recipients, Context, and Facilitation* and propagates understanding of the implementation and utilization of evidence-based practices in health systems (Kitson et al., 2008; Stetler, Damschroder, Helfrich, & Hagedorn, 2011). The Framework suggests that implementation and utilization is successful when the evidence is robust, the system of care is receptive to change, and the change process is appropriately facilitated.

## Methods

### Design

We utilized a mixed method design triangulating quantitative and qualitative methods (Bryman, 2006; Fetters & Freshwater, 2015) and the i-PARIHS Framework as conceptual background. The mixed method approach increased our understanding of factors influencing the implementation of evidence-based SC interventions for VLWH from the perspectives of clinicians. Our mixed method approach enabled a comprehensive view of the mechanisms influencing the utilization of evidence-based SC interventions within the VA setting.

### Setting

Veteran Affairs HIV clinics were purposively selected to provide a geographically diverse group that exists throughout the U.S. Sampling emphasis was placed on recruiting sites in the Southeastern area of the U.S. as this is the area with the fastest HIV infection rate (CDC, 2018b).

### Sample size determination

From a methodological perspective, prior research suggests that unique qualitative themes can be identified with 12 -15 participant individual interviews to achieve saturation (Guest, Bunce, & Johnson, 2006). Data triangulation process used in the study also enhanced saturation (Fusch & Ness, 2015).

### Participants and data collection process

Study procedures were approved by the Institutional Review Board of the investigators’ institution. Criteria for study eligibility included: (1) HIV clinicians operationalized as infectious disease fellows, nurse practitioners, and physician assistants licensed to provide direct care to VLWH at VA HIV clinics. We broadly operationalized ‘HIV clinicians’ in order to maximize our sample size and include a variety of viewpoints. Participants were excluded if they: (1) self-reported hearing loss that prevent phone interviews; (2) were not licensed to provide clinical care to VLWH; or (3) had status of a trainee (for example, intern or resident).

### Data collection process

After contacting Veterans Affairs HIV clinic directors at eight sites and providing the opportunity to review the study aims and research protocol, directors at all eight sites agreed to participate. As part of the recruitment process, directors were instructed by the research team to convey to potential participants (clinicians) that participation in the study was completely voluntary. Clinic directors gave clinicians permission to participate during regular working hours, and all clinicians working in participating sites were invited to participate. If an HIV clinician expressed interest in the study, the clinic director provided their name and contact information (VA email address and phone number) to the research team. The research assistant then contacted potential participants by email to provide more information and confirm their interest. After agreeing to participate, clinicians were scheduled for a phone interview and were sent study-related materials by email. Study-related materials included data collection forms (demographic form and survey instrument [described below]) and a VA form for obtaining permission to audio record the interviews. If no response was received from the participants after three days, the research assistant followed up with a phone call. Clinicians were given ample time to review the study materials and ask questions. Clinicians were asked to return the signed VA Consent Form by the day of the interview. During the interview, the research team reviewed the demographic form and survey, the Organizational Readiness to Change Assessment (ORCA), (Helfrich, Li, Sharp, & Sales, 2009) over the phone and recorded the clinician’s responses. After surveys were completed, the qualitative interview would begin. Survey data and recordings were labeled with a unique ID codes and only the study team had access to the key linking data to codes. Audio recordings were transcribed verbatim by a VA-approved transcription agency.

### Quantitative measures

#### Demographic assessment

The demographic assessment form included questions about demographic characteristics such as age, gender, education, length of employment in the VA, and smoking history.

#### Organizational readiness to change assessment (ORCA)

The ORCA was developed specifically for the VA setting, and is based on the i-PARIHS Framework. The tool was used to assess organizational readiness to adapt evidence-based interventions and organizational capacity to successfully implement tobacco cessation interventions. ORCA is a five-point Likert scale (e.g. 1 [*strongly disagree*] to 5 [*strongly agree*]). It has three sub-scales with a total of 75 items that correspond to the key elements of the i-PARIHS Framework and scores are summed for each subscale. The ORCA is well-validated and internal consistency for the sub-scales are: Evidence (*α *= 0.85), Context (*α* = 0.74), and Facilitation (*α *= 0.9) (Helfrich et al., 2009). It is important to note that because the ORCA was developed prior to recent updates to the PARIHS Framework, ORCA does not separately identify *Recipients* as conceptualized in the revised i-PARIHS, but incorporates *Recipients* in the constructs of *Evidence* (as patient preferences) and *Context* (staff culture). This difference was addressed by examining and reorganizing study results along the constructs of the new i-PARIHS Framework in all phases of the analysis (Table 1).

**Table 1.** i-PARIHS constructs with examples of corresponding qualitative and ORCA questions.

i-PARIHS Construct	Example Qualitative Questions	Example ORCA Items
Innovation	Please describe your perceptions of the need, or lack of need, for smoking cessation among HIV patients at your clinic. Tell me about what kind of background, experience, and training you might have with smoking cessation treatments? How does smoking cessation impact clinical processes?	Integrated smoking cessation treatment will improve health outcomes for patients with HIV. Integrated smoking cessation treatment for patients with HIV is supported by RCTs or other scientific evidence from VA.
Recipients	What do you hear patients say about the desire or need for smoking cessation? How successful do you think smoking cessation is among your patients? Could you please describe the extent to which, if at all, smoking cessation treatment occurs with HIV patients at your clinic?	Integrated smoking cessation treatment for patients with HIV are supported by clinical experience with VA patients. Integrated smoking cessation treatment for patients with HIV take into consideration the needs and preferences of VA patients. Staff members in your organization are receptive to change in clinical processes.
Context	What are the challenges of offering smoking cessation at your clinic? What are the things regarding smoking cessation that are working well at your clinic? What roles do departmental of facility leadership play in providing smoking cessation services?	Senior leadership/clinical management in your organization solicit opinions of clinical staff regarding decision about patient care. Senior leadership/clinical management in your organization promote communication among clinical services and units. In general in my organization, when there is agreement that change needs to happen we have the necessary support in forms of training/facilities/staffing.
Facilitation	Have you heard of or received a copy of the *HIV Provider Smoking Cessation Handbook*? What types of facilitation – in terms of focus and delivery strategies- would you prefer to receive? What are your colleagues’ facilitation needs?	Senior leadership/clinical management will provide clear goals for improvement in patient care. The implementation team members have staff support and other resources required for the project.

### Qualitative interviews

Qualitative interviews were conducted using a semi-structured interview guide developed by the research team. The purpose of the in-depth interviews was to elicit clinician perceptions and experiences with the utilization of SC interventions within the context of HIV care in the VA. Topics included types of SC available at the providers’ clinic, the perceived need for offering SC to VLWH, and providers’ thoughts and experiences with patient readiness and follow-up on SC referrals. Questions were designed to reflect the key elements of the i-PARIHS Framework and integrating the ORCA questions (Table 1). For example, questions were asked related to participants’ perceptions of the current and anticipated barriers and enablers to utilizing SC treatments among VLWH, personal experiences when offering SC care, and observations of SC initiatives conducted by other clinicians. Questions were also asked about how shifting the structural and organizational landscape of VA healthcare might facilitate or impede SC efforts.

## Qualitative data collection process

The semi-structured interviews were conducted by one of two senior members of the research team (the first author and another doctoral-level researcher). In preparation for the interviews, all members of the research team jointly reviewed the principles of qualitative interviewing and ran through several mock interviews (Seidman, 2006). The interviews lasted approximately 30-45 min. Participants were interviewed during regular working hours and thus were not compensated in accordance with federal employment guidelines.

## Data analyses

### Quantitative data analysis

Data from the ORCA were analyzed using descriptive statistics with means and standard deviations presented in Table 2. The answers to the questions complemented the qualitative analysis.

**Table 2.** Organizational readiness to change assessment data.

Scale	Subscale	Mean (SD)
Evidence/Innovation		4.24 (0.36)
	Research	4.41 (0.41)
	Patient Preferences	4.06 (0.48)
	Clinical Experiences	3.91 (0.63)
Context		3.81 (0.56)
	Staff Culture	4.13 (0.58)
	Opinion Leaders	4.07 (0.44)
	Measurement	3.94 (0.68)
	Leadership Culture	3.82 (0.84)
	Leadership	3.72 (0.96)
	Resources	3.11 (0.93)
Facilitation	3.85 (0.45)	
	Project Evaluation	4.19 (0.50)
	Implementation Plan	4.00 (0.56)
	Project Communication	3.92 (0.59)
	Clinical Champion	3.89 (0.42)
	Project Progress Tracking	3.86 (0.45)
	Leaders’ Practices	3.80 (0.51)
	Leadership Implementation Roles	3.76 (0.65)
	Project Resources	3.73 (0.65)
	Implementation Team Roles	3.67 (0.67)

Note: ORCA answer responses range from 1=strongly disagree to 5=strongly agree.

### Qualitative data analysis

Transcripts were proofread and de-identified prior to analysis. Preliminary qualitative analysis was conducted by three of the authors (SH, AP, and AF) and a doctorally-prepared VHA researcher. SH and AF were trained by AP and the VHA researcher who have extensive experience in conducting qualitative research and analysis. Weekly meetings provided the platform to discuss and reconcile divergent results. A summary table with emergent themes was created utilizing standard word processing software. The summary table allowed for the reduction of data without the loss of key information (Miles & Huberman, 1994), and included key domain headings and definitions as a codebook to provide structure. For example, the domains of ‘Need’ and ‘Patient Experience’ were selected deductively using the i-PARIHS Framework; other domains were identified inductively to address emerging themes (the list of domains can be made available upon request). During the development of the summary table, we independently and thoroughly summarized each transcript and then met as a group to compare completed summaries for similarities and differences. Differences were reconciled through an iterative process. This process was repeated to check for consensus and to finalize the summary table. Individual researchers’ transcript summaries were then reconciled into a ‘master’ summary for each transcript. After the first seven transcripts were summarized, the data from the master summary templates were copied onto a Microsoft Excel matrix to begin analysis and identification of common themes within and across participants and sites (Miles & Huberman, 1994). We met regularly to ensure ongoing consensus and discussion of the domains, themes, and findings allowing the research team to update the interview guide, the summary domains, and the analysis process to reflect new questions and themes as they were identified from the data. Additionally, VNC who has experience with qualitative and mixed method designs was invited by SH to participate and she contributed to identifying the themes in the final stages of the analysis process.

### Mixed method integration

Findings from the quantitative and qualitative were integrated through the process of triangulation. Triangulation is ‘the checking of inferences drawn from one set of data sources by collecting data from others,’ especially in cases of ‘data produced by different data collection techniques’ (Hammersley & Atkinson, 2007, p. 183).

## Results

### Description of demographic characteristics

Nineteen clinicians working at eight different facilities across the VA system in the United States participated in the study and ranged from one to three clinicians per facility. Ten (N = 19) were from facilities representing the Northeast and Mid-Atlantic regions, while the remainder (9/19) were from facilities representing the South and South West regions. Participants worked at the VA for an average of 12 years (range 5 months – 34 years), and the vast majority were physicians (14/19). Most clinicians self-identified as being white (15/19), non-Hispanic (17/19), men (10/19), and never smoked (19/19). See Table 3 for a complete list of participants’ demographic characteristics.

**Table 3.** Respondent demographic characteristics (*N* = 19).

Variable	N	%
*Gender*		
Male	10	52.6
Female	9	47.5
Clinical job position*		
Nurse Practitioner	3	15.8
Physician /PA	16	84.2
*Education Level*		
≤ Graduate degree*	5	26.4
Medical degree	14	73.7
*Race*		
White	15	78.9
POC*	4	21.1
*Ethnicity*		
Hispanic	2	10.5
Non-Hispanic	17	89.5
*Smoking Status*		
Not a smoker	19	100
Age (M ± SD)	47.3 ± 13.5	
Length of time employed by VA (M ± SD)	12.8 ± 12.2	

POC = people of color; VA = veteran’s affairs.

*Categories combined due to small values and to maintain participant confidentiality.

## Themes

Seven themes emerged from the interviews. While we used i-PARIHS to start our analysis (deductively identifying constructs for the summary table), we also took an inductive approach to analyze interviews data guided by qualitative method. Therefore, our results are organized and presented according to the seven themes that best fit the data and not necessarily what suited i-PARIHS. Themes and the relationships with the i-PARIHS Framework constructs of *Innovation, Recipients, Context,* and *Facilitation* are discussed to introduce the themes*.* Clinician status and years as a VA employee are provided in parentheses following exemplar quotes.

## Innovation construct

*The initial construct of Evidence which includes the adoption of* sources of evidence to meet the unique priority needs of participants in specific contexts was extended in the i-PARIHS Framework. The notion of blending sources of evidence, clinical practice, patients’ needs, and preferences are integral to this construct. The Innovation construct was added to capture the application of SC evidence and its adoption or ‘uptake’ by VLWH (i.e. usability), particularly in the domains of clinicians’ perceived need for SC interventions for VLWH that smoked. We also included clinicians’ view of patients’ experiences and adoption of SC interventions.

### Theme 1: perceived usability for smoking cessation as a priority need

Clinicians emphasized that SC is essential for VLWH and viewed as a priority need as the use of ARV increases life expectancy. Additionally, VLWH are at increased risk for age- and tobacco-related illnesses compared to non-HIV populations.

When you think of all the major aging type issues and cancers that HIV patients will develop at earlier ages or will develop at higher rates than the non-HIV patients, you put tobacco in there as a causative agent in almost all of them. I mean I don’t know that we put as much emphasis on it as we should, but I think under medication I think probably one of the top most helpful things we could do is to do a good job at smoking cessation. (Physician’s Assistant, 25yrs)

Clinicians reported that SC was one of many shifting priorities for patients. If a patient has other urgent HIV care needs such as substance abuse, or other medical conditions, then SC would be relegated to a lower priority. One notable exception was if patients presented with smoking-related conditions, such as respiratory infection or other cardiovascular risk factors, then SC is prioritized.

But if I'm seeing somebody in urgent care who's got an upper respiratory infection for the fourth or fifth time this year then I'll take the lead and push a little harder and say, you know, we've got a bunch of [SC] options that we can offer you … . (Nurse Practitioner, 20 yrs)

### Theme 2: knowledge, attitudes, and confidence to implement SC

Knowledge sources, existing values, and the degree to which these are integrated in the practice contexts are also important aspects of the Innovation construct. Clinicians expressed varying levels of comfort when discussing and treating VLWH. Clinicians new to the VA reported feeling uncomfortable, often citing lack of knowledge of best practices, training, and the availability of SC alternatives to nicotine replacement therapy (NRT). Some clinicians had no formal training in SC and identified it as a critical training need. They were generally more comfortable with NRT compared to other SC interventions.

I don't think I've had any specialized training in smoking cessation treatment. I think what I … learned about different approaches is just what I learned in residency … and really doesn't involve much other than nicotine patches and gum. (Infectious Disease MD, 5 months)

### Recipient construct

The *Recipient* construct captures patient motivation to accept support for the implementation and utilization of SC interventions. Motivations are informed by patient views, beliefs, and experiences with established practices, which drive the successful implementation of SC interventions.

### Theme 3: perceptions of patient experiences and active engagement

Clinicians reported that patients’ readiness and motivation to quit was important and unless patients express interest in quitting, it will be ineffective and may affect the clinician-patient encounter.

Involving the patient is always the first step … If there is no patient buy-in … I don’t think there is much of a chance for being successful. (Infectious Disease MD, 11yrs)

Clinicians also reported the need for easy-to-implement strategies to prevent losing motivation if patients expressed interest. Patients were perceived as unwilling to attend extra appointments at the VA to continue with the SC treatment. Time, support, and resources are important characteristics of the Recipient construct as captured in the words from a nurse practitioner.

Most of the time, because of time commitments, patients usually just–opt [out] … because they sometimes have concerns about time commitments. (Nurse Practitioner, 8 months)

Clinicians report that VLWH acknowledge that they should quit, but not all are ready to do so, with many patients reducing smoking rather than quitting. Competing priorities (e.g. social problems, other addictions) are perceived to influence patients’ motivation for SC. Several clinicians noted that smoking may be seen as a ‘crutch’ to help patients deal with problems in their everyday lives.

… have too many other things in their lives … A lot of them are homeless. A lot of them don't have enough money to live on. And they worry about day-to-day things that keep them alive. And smoking is like a crutch they got to lean on when things get tough. (Infectious Disease MD, 34 yrs)

### Context construct

The *Context* construct conceptualizes readiness for SC implementation and utilization that focuses on inner (immediate local setting) and outer (policy, social, political, and regulation infrastructure) contexts at the micro, meso, and macro levels of function. *Context* was evaluated as barriers and enablers to providing SC interventions, which are related to resources, culture, leadership evaluation, interest, attitudes toward learning for change, and learning environment.

### Theme 4: barriers to SC utilization

Clinicians identified the lack of SC education, knowledge, and skills as major barriers to utilizing SC efforts. Also, structural barriers such as limited time, space to conduct SC training, and grant funding to facilitate utilization were identified.

One of the challenges about approaching patients with smoking cessation, is that you really need time to finish the whole conversation with them. If you're just going to say to them how'd you like to quit smoking, that's not going to mean very much to them. So in order to have the entire conversation that's meaningful, I think it takes time. Probably takes about 10, 15 min to get the entire thought process through, what the risks are, and that they're much better off not smoking. … if you got eight patients in clinic that morning, or 12 patients that morning, that you're really willing to spend 15 min, or the whole conversation, rather than just do the perfunctory thing and say well, are you interested in stopping smoking? No? Okay. And just move on. You've got other patients waiting. … (Infectious Disease MD, 34 yrs)

There is also variability in individual clinicians’ SC efforts; a lack of clear and consistent communication between clinicians about SC efforts prevents coordinated efforts. While some clinicians had SC resources on-site (e.g. social workers), other clinicians identified a lack of coordinated SC efforts as a barrier.

I think a barrier for the patient … is that our pharmacy wants all of these [SC prescriptions] to be mailed out and I think sometimes we may miss an opportunity for somebody who's … on the fence about smoking cessation, or they're willing to quit today because their bronchitis is so bad they can't breathe anyway and so they've already quit for 48 h. … , by the time they actually get whatever they're going to use they're feeling better and they're back to smoking. (Nurse Practitioner, 20 yrs)

Without on-site integration of SC efforts, it is difficult for clinicians to evaluate the success of SC among patients.

### Theme 5: enablers to SC utilization

Important contextual enablers to SC utilization were identified. Clinicians with on-site pharmacy services noted that the ease of prescribing NRT and/or other pharmaceuticals is helpful. Having on-site classes and social workers to review SC options with patients are enablers to SC.

We're fortunate enough to have two social workers with us in [the]clinic, so they're right here … actively integrated into patient care. Certainly if she gets backed up on a particular day she may sort of touch base with the person and then setup a separate appointment … (Nurse Practitioner, 20 yrs).

Many clinicians reported taking a person-centered supportive approach. Not all patients would respond to the same type of treatment; thus, substantiating the importance of personalized care for SC.

### Facilitation construct

The *Facilitation* construct includes the facilitator role (individuals and teams) and facilitation process (strategies and techniques) that enable and activate the utilization of SC. Facilitation role or facilitators are individuals or teams that ensure that strategies are modified for maximum uptake or adoption in tandem with the necessary collaborative engagement of institutional networks.

### Theme 6: need for resource utilization

To evaluate the facilitation process or strategies, we were particularly interested in if clinicians received or used the *HIV Provider Smoking Cessation Handbook* and the accompanying patient workbook, *My Smoking Cessation Workbook: A Resource for Patients,* and/or were referring patients who smoke to VA-sponsored telephone-based quit lines. Some clinicians reported that they neither used nor heard of the VA quit lines or Handbook and some had heard of but did not use them.

I think I had heard that. I haven’t specifically referred any patients to that or didn’t necessarily know the details of the program. (Infectious Disease MD, 22 months)

### Theme 7: need for more facilitators and facilitation

Clinicians largely agreed that on-site SC champions would be effective. They could mitigate barriers to SC utilization. Greater involvement and engagement of clinicians and staff and a formalized peer support for SC were also suggested to improve SC efforts.

Well, I think it could be sort of a team effort. It might be a combination of providers such as the nurse practitioners and … partnerships between nurse practitioners and some of the mental health staff. (Nurse Practitioner, 8 months)

Clinicians suggested that it would be important to reinforce current policies and procedures that prohibit smoking in VA facilities. Additionally, they reported that these efforts would spotlight the importance of SC and a high priority for VLWH.

## Quantitative results

On the entire ORCA scale, clinicians agreed to strongly agreed (*M* = 4.5, *SD* = 0.4) that SC interventions will improve health outcomes for VLWH who smoke. Clinicians also agreed to strongly agreed (*M* = 4.2, *SD* = 0.4) to the utilization of SC interventions within the HIV clinical setting. With respect to the ORCA sub-category of Context, on average clinicians neither agreed nor disagreed (*M* = 3.8, *SD* = 0.6) that the VA supports the utilization of SC in HIV clinical settings. Additional concern was noted regarding having enough staff, training, space and funding to utilize smoking cessation interventions (*M* = 3.1, *SD *= 0.9). Related to the ORCA sub-category of Facilitation, on average participants neither agreed nor disagreed to agreed (*M* = 3.8, *SD* = 0.4) that there was adequate facilitation to utilize SC interventions.

## Data triangulation

Results from the quantitative ORCA results converged with the qualitative interviews. Specifically, clinicians felt a high degree of confidence in the construct of *Evidence, with* the highest mean score and items based on the utilization of best evidence, clinician expertize, and patient preferences and values. In the qualitative interviews, participants were particularly concerned about the level of priority placed in SC. Additionally, there was limited training available to some clinicians to address the knowledge gaps related to the SC for VLWH (innovation). *Facilitation* was the second highest mean score focused on the characteristics of the team members and process strategies and techniques. Participants reported an enormous need for resources, trained clinicians, and system-wide strategies for successful implementation and utilization. Lastly, *Context* included items on resources, barriers, and enablers. Table 2 has detailed descriptions of the items included in the ORCA. In the qualitative interviews, context was addressed in the form of barriers and enablers in terms of time to dedicate to SC program-related discussions, private rooms to hold the discussions, and the availability of multidisciplinary teams, and the need for champions or navigators to attend to the multilevel factors of SC for VLWH. Efforts to triangulate the results from the two methodological approaches converged; thus, enhancing validation and clarification of the SC interventions for veterans with HIV infection (Bazeley, 2009; Bryman, 2006).

## Discussion

The aim of the study was to evaluate factors influencing the utilization of SC interventions to adopt evidence-based interventions using a mixed method approach to triangulate findings and understand the mechanisms associated with the i-PARIHS Framework (i.e. *Innovation, Recipient, Context, Facilitation*) in the implementation and utilization of evidence-based SC interventions in VA clinic settings. Overall, clinicians agreed that there was strong evidence that smoking was related to greater morbidity and mortality among VLWH. Clinicians were more interested in utilizing SC interventions, while specialists thought SC was an issue better managed by the veteran’s primary care team. In a systematic review of SC in adults with substance abuse treatment or recovery conducted by Thurgood, McNeill, Clark-Carter, and Brose (2016), investigators found that behavioral support by clinic staff in conjunction with NRT was integral in facilitating SC (Thurgood et al., 2016). They reported that the individualized counseling prompted SC maintenance and prevented relapse. Another study on SC in women living with HIV found that a configuration of behavioral support, motivational interviewing, combined with NRT as opposed to prescribed advice alone decreased the mean cigarettes smoked per day (Manuel et al., 2013). Video group SC interventions for PLWH have promise, particularly for those who prefer virtual participation for convenience. Designing a population-specific, client-centered intervention would be more successful because this population has several influencers beyond physiological desire such as the social and emotional contexts (Ignacio et al., 2018; Shiltz, Paniagua, & Hastings, 2011; Thurgood et al., 2016; Wilson et al., 2017). Studies substantiate the importance of a combination of strategies and the dedicated time and effort required for successful implementation of SC intervention programs (West, 2017).

HIV clinicians identified several important contextual themes related to the i-PARIHS Framework that substantially limited the reach and impact of the utilization of SC efforts at the VA. Lack of education and experience using evidence-based SC interventions was noted as a consistent barrier. Compounding the lack of training was frustration with the perceived lack of interest of patients in quitting, and poor outcomes attributed to current SC interventions. Most reported a strong interest in receiving additional training to enhance knowledge and competence to utilize SC interventions effectively. These important perspectives provide the opportunity for targeted interventions to improve the implementation and adoption of SC programs for VLWH.

Contextual barriers to providing SC treatment were also identified. There were mixed views regarding electronic record reminders for SC screening. Some clinicians used the reminders to begin the discussion on SC while others ignored them. Clinicians reported that a lack of dedicated time and space limited the utilization of on-site SC interventions. Utilization was also hampered by the variable access to facility-level SC treatment to which they could refer their patients. Among clinicians reporting the ability to refer to facility-level SC treatment, most expressed concerns that the times and locations of these interventions were not readily accessible to working veterans or those traveling long distances. The vast majority of clinicians were unaware that the VA supported free, telephone-based SC quit lines. Our study was consistent with other studies (Bean et al., 2016; Cunningham, Kaboli, Ono, & Vander Weg, 2011; Ledgerwood & Yskes, 2016; Thurgood et al., 2016). The literature on VLWH is sparse. However; compared to a study conducted by Ledgerwood and Yskes (2016), SC among PLWH was emphasized but there were barriers to proper utilization of evidence-based interventions. These barriers included patient resistance, insufficient counseling time, and lack of provider confidence. Another area of successful utilization was the length of time invested in SC. A longitudinal study conducted by Burns, Rothman, Fu, Lindgren, and Joseph (2014) found that individuals who received long-term follow-up care were more likely to remain abstinent than those who were offered routine care suggesting the need for rigorous and longitudinal studies for SC among VLWH.

Facilitation (as a role and process) was perceived by most of the clinicians as an aspirational ideal, but with important opportunities. Clinicians largely agreed that on-site SC champions would be an effective strategy to overcome contextual barriers to SC but funding for such positions is limited. Only one of the eight sites reported having access to an integrated SC service within the HIV clinic setting led by a social worker who provided evaluations, behavioral treatment interventions, and served as a facilitator at the site. Clinicians affiliated with the site were satisfied with this service delivery framework.

## Implications

Harris (2010) identified that a majority of the literature in existence on this subject matter is geared towards discovery research rather than delivery or implementation research (Harris, 2010). This indicates opportunities to investigate best approaches in the utilization of SC among VLWH given the paucity of empirical evidence. Gaps can be addressed through the collaboration of researchers and practitioners and the continued reporting of challenges faced by practitioners for iteration of programs (Harris, 2010). Furthermore, given the numerous barriers identified in our study, efforts to strengthen the enablers such as funding for SC facilitators and clinician champions are warranted.

Integrating smoking cessation programs into HIV treatment plans in the veteran patient population is critical. A coordinated effort between the VA central office and VA facilities may be needed to ensure the training of clinicians and staff on the practical use of evidence-based approaches for SC among VLWH. Given the morbidity and mortality associated with smoking among VLWH, it may be important to evaluate whether facility level financial incentives directed specifically at implementing SC interventions may be needed. It is clear that dedicated time and effort will maximize this effort and yield successful utilization and subsequently facilitate patient compliance. Importantly, a combination of strategies such as motivational interviewing, NRT, and informational sessions for sustainability have shown success. In addition, implementation of long-term interventions may be more useful than occasional one-time interventions because long-term follow up care reinforces abstinence and provides a safeguard against relapse.

## Study limitations

The study results may only be generalizable to patients with similar demographic characteristics. Additionally, there was limited statistical power with the aim to test hypotheses and not to find significance.

## Conclusions

Smoking is prevalent in the veteran population due to factors that incentivize tobacco use such as lower cost of tobacco products on military bases, dedicated time for smoke breaks at work, and smoking as an idle activity or stress reliever. Even though there is ample evidence supporting the association of increased morbidity with smoking among VLWH, SC is less emphasized. The convergence of the ORCA results and identified themes underscore the need for clinicians working in VA HIV clinics to provide SC training and supportive care to motivate VLWH to stop smoking. Working toward ways to better facilitate the implementation and utilization of SC programs for VLWH would be an important next step for the VA system of care.

## Data Availability

Data for the results in the manuscript can be available from the first author upon request.
